# Scientific instrument Femtosecond X-ray Experiments (FXE): instrumentation and baseline experimental capabilities^1^

**DOI:** 10.1107/S1600577519006647

**Published:** 2019-08-09

**Authors:** Andreas Galler, Wojciech Gawelda, Mykola Biednov, Christina Bomer, Alexander Britz, Sandor Brockhauser, Tae-Kyu Choi, Michael Diez, Paul Frankenberger, Marcus French, Dennis Görries, Matthiew Hart, Steffen Hauf, Dmitry Khakhulin, Martin Knoll, Timo Korsch, Katharina Kubicek, Markus Kuster, Philipp Lang, Frederico Alves Lima, Florian Otte, Sebastian Schulz, Peter Zalden, Christian Bressler

**Affiliations:** a Femtosecond X-ray Experiments Group, European XFEL, Holzkoppel 4, 22869 Schenefeld, Germany; b Faculty of Physics, Adam Mickiewicz University, Umultowska 85, 61-614 Poznań, Poland; c The Hamburg Centre for Ultrafast Imaging, Universität Hamburg, 22607 Hamburg, Germany; d Biological Research Centre (BRC), Hungarian Academy of Sciences, Temesvári krt 62, H-6726 Szeged, Hungary; e STFC Technology, Rutherford Appleton Laboratory, Didcot, Oxfordshire OX11 0QX, UK; f Fakultät Physik, Technische Universität Dortmund, 44227 Dortmund, Germany

**Keywords:** XFELs, X-rays, pump–probe, ultrafast science

## Abstract

The European X-ray Free-Electron Laser (EuXFEL) delivers extremely intense (>10^12^ photons pulse^−1^ and up to 27000 pulses s^−1^), ultrashort (<100 fs) and transversely coherent X-ray radiation, at a repetition rate of up to 4.5 MHz. Its unique X-ray beam parameters enable novel and groundbreaking experiments in ultrafast photochemistry and material sciences at the Femtosecond X-ray Experiments (FXE) scientific instrument. This paper provides an overview of the currently implemented experimental baseline instrumentation and its performance during the commissioning phase, and a preview of planned improvements. FXE’s versatile instrumentation combines the simultaneous application of forward X-ray scattering and X-ray spectroscopy techniques with femtosecond time resolution. These methods will eventually permit exploitation of wide-angle X-ray scattering studies and X-ray emission spectroscopy, along with X-ray absorption spectroscopy, including resonant inelastic X-ray scattering and X-ray Raman scattering. A suite of ultrafast optical lasers throughout the UV–visible and near-IR ranges (extending up to mid-IR in the near future) with pulse length down to 15 fs, synchronized to the X-ray source, serve to initiate dynamic changes in the sample. Time-delayed hard X-ray pulses in the 5–20 keV range are used to probe the ensuing dynamic processes using the suite of X-ray probe tools. FXE is equipped with a primary monochromator, a primary and secondary single-shot spectrometer, and a timing tool to correct the residual timing jitter between laser and X-ray pulses.

## Abstract 2

The Femtosecond X-ray Experiments scientific instrument is a versatile instrument for ultrafast pump–probe X-ray absorption, emission and scattering experiments at the European XFEL; the instrumentation, commissioning results and operation performance status are presented.

## 1. Introduction   

More than three decades ago coherent molecular dynamics were observed in ‘real time’ on a picosecond timescale exploiting short-pulse lasers (Lambert *et al.*, 1981 ▸). Since then, an impressive development of ultrafast optical spectroscopies has taken place (Zewail, 2000 ▸), addressing the timescale of atomic movements from picoseconds (ps) to femtoseconds (fs), *i.e.* from 10^−12^ to 10^−15^ s, respectively, and already entering the attosecond (as) time domain (Calegari *et al.*, 2016 ▸; Gallmann *et al.*, 2012 ▸). Fundamental processes in physics, chemistry and biology occur in this ultrashort time range, exemplified by the timescale of chemical bond breaking, formation and structural reorganization, by the transfer of energy and charge through molecules and solids, and by changes of spin states and guest–host interactions. In other words, with the advent of fs optical tools we have gained a deeper understanding of the underlying elementary steps in all kinds of matter.

Despite the ongoing success of femtochemistry (Zewail, 2000 ▸), some fundamental questions regarding ultrafast processes remain unsolved. Optical domain spectroscopy does not directly deliver evolving structural information and in consequence efforts have been undertaken to combine the high time resolution of fs lasers with the high spatial resolution (at the atomic level) of structurally sensitive tools using electrons or X-rays (Chergui & Zewail, 2009 ▸). The next step aimed at pushing the timescale of structure-sensitive techniques into the fs region, and this expanded towards element-sensitive structural tools capable of directly resolving the electronic, spin and geometric structure changes during the course of a chemical reaction and their influence on the chemical reactivity.

With the construction of ultrafast short-wavelength light sources over the past ten years, *i.e.* free-electron lasers (FELs), which extend the available photon energies from soft to hard X-ray ranges (0.1–20 keV), entirely new experiments in ultrafast photochemistry have been made possible (Ullrich *et al.*, 2012 ▸; Gallmann *et al.*, 2012 ▸; Ackermann *et al.*, 2007 ▸; Emma *et al.*, 2010 ▸; Shintake *et al.*, 2008 ▸). In particular, FEL sources, based on the self-amplified spontaneous emission (SASE) process, have become operational at several large-scale facilities worldwide and provide intense coherent vacuum UV to X-ray radiation with fs pulse lengths (Emma *et al.*, 2010 ▸; Shintake *et al.*, 2008 ▸). A multitude of experiments have successfully demonstrated that this new generation of light sources provides unprecedented insight into structural dynamics in various condensed-matter systems (Chergui & Collet, 2017 ▸; Alonso-Mori *et al.*, 2015 ▸; Abela *et al.*, 2017 ▸; Chollet *et al.*, 2015 ▸; Clark *et al.*, 2013 ▸; Först *et al.*, 2013 ▸; Chen *et al.*, 2016 ▸). Particularly relevant for the scientific scope of the Femtosecond X-ray Experiments (FXE) instrument are studies focused on tracking correlated electronic and nuclear motion in a strongly nonadiabatic regime during the formation and breaking of chemical bonds, which aim at filming the nuclear motions during chemical reactions with atomic spatial and temporal resolution while tracking the excited-state dynamics of reacting molecules (Mara *et al.*, 2017 ▸; Miller *et al.*, 2017 ▸; Lemke *et al.*, 2017 ▸; Zhang *et al.*, 2014 ▸, 2017 ▸; van Driel *et al.*, 2016 ▸; Shelby *et al.*, 2016 ▸; Biasin *et al.*, 2016 ▸; Pande *et al.*, 2016 ▸; Barends *et al.*, 2015 ▸; Kern *et al.*, 2015 ▸; Zhang & Gaffney, 2015 ▸; Kim *et al.*, 2015 ▸; Canton *et al.*, 2015 ▸; Levantino *et al.*, 2015 ▸; Arnlund *et al.*, 2014 ▸).

Since 2017, the European X-ray Free-Electron Laser (EuXFEL) user facility has offered X-ray pulses with the world’s highest average brilliance due to its superconducting accelerator that allows for a remarkably high 4.5 MHz repetition rate pulse pattern – and its delivery of up to 2700 intense, ultrashort, transversely coherent X-ray pulses per second in 10 Hz bursts (Altarelli *et al.*, 2006 ▸), or up to 27000 pulses s^−1^, which is more than a hundredfold what other FEL facilities can offer. In combination with advanced scientific instrumentation and sample preparation techniques, this unmatched average X-ray flux opens unprecedented opportunities for the investigation of time-dependent phenomena, such as solution-phase photochemistry (Mara *et al.*, 2017 ▸; Miller *et al.*, 2017 ▸; Lemke *et al.*, 2017 ▸; Zhang *et al.*, 2014 ▸, 2017 ▸; van Driel *et al.*, 2016 ▸; Shelby *et al.*, 2016 ▸; Biasin *et al.*, 2016 ▸; Pande *et al.*, 2016 ▸; Barends *et al.*, 2015 ▸; Kern *et al.*, 2015 ▸; Zhang & Gaffney, 2015 ▸; Kim *et al.*, 2015 ▸; Canton *et al.*, 2015 ▸; Levantino *et al.*, 2015 ▸; Arnlund *et al.*, 2014 ▸) and material science studies on crystalline and non-crystalline samples (Hruszkewycz *et al.*, 2012 ▸; Glover *et al.*, 2012 ▸; Brown *et al.*, 2015 ▸; Ferrer *et al.*, 2015 ▸; Gerber *et al.*, 2015 ▸; Jiang *et al.*, 2016 ▸; Kozina *et al.*, 2017 ▸; Mannebach *et al.*, 2017 ▸). One example is hard X-ray Raman scattering (XRS): one can seek to use every single X-ray pulse at 4.5 MHz to collect the required statistics, thus obtaining the information content of soft X-ray spectra while maintaining the experimental benefits of hard X-ray techniques (Bergmann *et al.*, 2002 ▸; Szlachetko *et al.*, 2017 ▸). Moreover, the high flux enables time-resolved studies on very dilute solutions, a typical challenge for biologically relevant systems. The scientific instrument FXE is designed to advance into this uncharted territory, utilizing hard X-rays to make fs-resolved ‘molecular movies’ of ensuing nuclear and electronic dynamics while exploiting the facility’s uniquely high average flux (Bressler *et al.*, 2012 ▸).

## 2. Overview of the scientific instrument FXE   

The FXE instrument is located at the end of the 915 m-long SASE1 beamline and consists of a dedicated undulator section and X-ray photon transport tunnel to guide the X-ray beam to the experimental hall (see Section 3 for a detailed description). The end-station successfully started user operation in September 2017. Similar to other instruments focused on time-resolved studies at existing X-ray FEL (XFEL) sources (Chergui & Collet, 2017 ▸; Alonso-Mori *et al.*, 2015 ▸; Abela *et al.*, 2017 ▸; Chollet *et al.*, 2015 ▸), FXE uses ultrafast optical laser pulses to generate transient states while the X-ray pulses probe their dynamical evolution. The FXE optical laser is a dedicated high-power system that has been developed to serve the EuXFEL facility (see Section 5 for details): it is synchronized to the unique X-ray pulse train pattern (burst mode) and can also accommodate the highest repetition rate of 4.5 MHz in burst mode (Palmer *et al.*, 2019 ▸). Thus, the laser pulse pattern follows the time structure of X-ray pulses delivered by the XFEL. The laser system is also tunable across the UV–visible up to near-IR range and delivers 15 fs-long pulses at 800 nm. In addition, a commercial fiber-based MHz laser system is available, but with less pulse intensity (20 µJ at 1030 nm) and longer pulse widths (∼300 fs FWHM).

For probing the transient structures, a versatile suite of instrumentation with different and complementary X-ray techniques is available. A dispersive X-ray emission spectrometer, based on a von Hamos design previously implemented at the Linear Coherent Light Source (Alonso-Mori *et al.*, 2015 ▸), can be utilized in combination with different X-ray detectors, operating at either 10 Hz (inter-train) or sub-MHz (intra-train) repetition rates. These include CCD cameras (GreatEyes Company), the Jungfrau 2D (with 75 µm pixels) (Redford *et al.*, 2018 ▸) or the Gotthard 1D strip (50 µm pitch) detectors (Mozzanica *et al.*, 2012 ▸). The von Hamos spectrometer allows for X-ray emission spectroscopy (XES) tracking of ultrafast changes in the electronic structure around a selected atom in the sample by recording the corresponding X-ray emission spectra (thus element-specific with fs time resolution). For X-ray solution scattering (XSS) or X-ray diffraction (XRD), which are the dominant techniques for probing concomitant structural changes, the Large Pixel Detector (LPD) (Hart *et al.*, 2012 ▸) is used. Further instrumentation, as described below, allows use of the X-ray absorption spectroscopy (XAS) and inelastic X-ray scattering (IXS) techniques, including in the future XRS, for structural dynamics research. In comparison with similar time-resolved end-stations at other XFEL facilities, the main differences in instrumentation concern the materials and concepts used for design of the beamline components, which have to withstand the high heat load induced by the intense X-ray beam. Furthermore, substantially faster sample delivery/exchange is implemented to operate reliably with the newly developed optical pump laser at X-ray pulse repetition rates of up to 4.5 MHz (Altarelli *et al.*, 2006 ▸). In this article the scientific instrument FXE and its current performance status are presented in detail.

## 3. X-ray beam transport and X-ray pulse structure   

The SASE1 undulator serves as the source of hard X-ray radiation (Sinn *et al.*, 2019 ▸). Its variable gap structure makes it possible to generate 5–20 keV radiation without changing the electron beam energy. Up to 35 undulators, each 5 m long (and with a 4 cm pole period and *K* parameters of 1.65–3.9), can be inserted to drive the stimulated emission into saturation (Abeghyan *et al.*, 2019 ▸). Currently, the source provides 1–1.5 mJ pulse energy at 9.3 keV, but the design goal will eventually cover all energies in the 5–20 keV range, with pulse energies above 4 mJ expected (Abeghyan *et al.*, 2019 ▸; Grünert, 2019 ▸).

**Figure 1:**
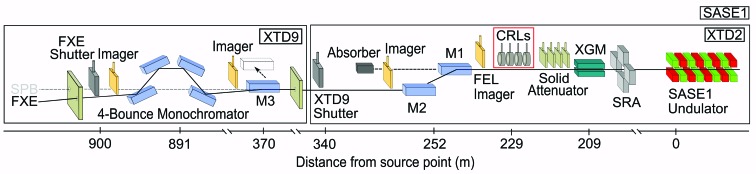
The X-ray beam transport system from the undulator exit (at the right) to the experiment hutch of the FXE instrument (on the left) spans over 900 m. It includes three mirrors: two offset mirrors reject higher-energy synchrotron radiation (M1 and M2), and one deflection mirror (M3) guides the beam into the FXE beam path (the straight beam path with retracted M3 guides the beam to the neighboring Single Particles, clusters, and Biomolecules & Serial Femtosecond Crystallography (SPB/SFX) instrument (Mancuso *et al.*, 2019 ▸). Compound refractive lenses (CRLs) collimate the beam to a beam size of 1–2 mm (depending on the X-ray energy). Beam imagers and a gas-based intensity position monitor (XGM) allow monitoring of the beam profile and location. Further components include the synchrotron radiation aperture (SRA), solid attenuator and, behind the offset mirror M2, an absorber to reject transmission of high-energy synchrotron radiation.

The X-ray beam exits the undulator with a divergence of around 2 µrad (at 10 keV), which delivers a fluence at the exit that is too large for any X-ray optics to withstand the high heat load. This is only possible more than 200 m from the source, where consequently the first optical elements in the tunnel are installed: 230 m from the undulator exit a ten-element compound refractive lens (CRL) system for beam collimation is placed, which allows the X-ray beam to be fully placed on the three mirrors along the beam path (especially the most distant one, M3, see Fig. 1 ▸), and thus to transport the entire available X-ray flux into the FXE experiment hutch.

Imagers along the beam path allow the beam position and profile to be monitored over the entire tunnel length (Grünert, 2019 ▸). The X-ray beam is guided through both offset mirrors and one deflection mirror (Sinn, 2019 ▸) into the optics branch of the FXE instrument. At the end of the photon transport tunnel, the front-end shutter of FXE is located right before the FXE experiment hutch.

**Figure 2:**
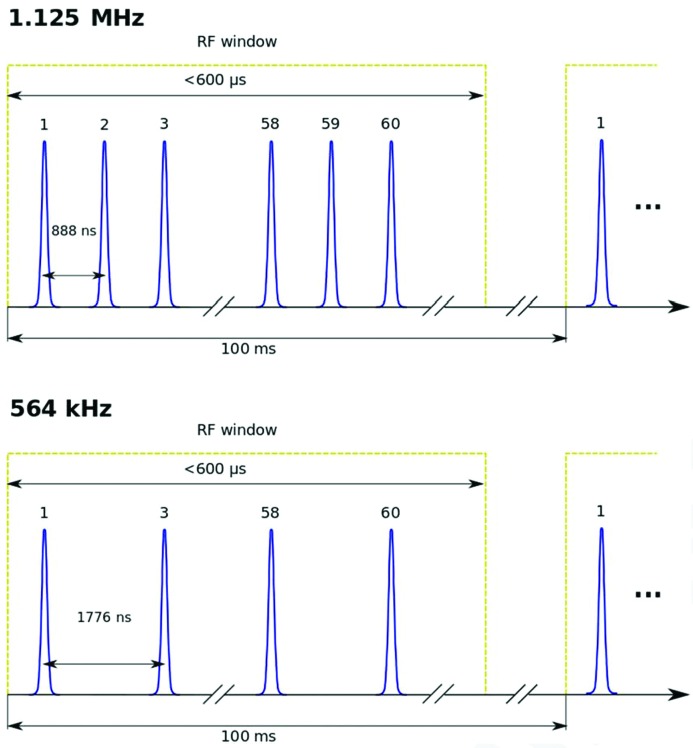
Pulse train filling pattern at two different intra-train repetition rates. Currently, the repetition rate is 1.125 MHz, or one pulse every ∼888 ns, with a maximum of 120 bunches per pulse train. Lower intra-train repetition rates are obtained by removing electron bunches from a train, thus reducing the total number of pulses to obtain the lower repetition rate at the experiment.

The X-ray pulses delivered by the EuXFEL are not evenly distributed but follow a structure of pulse trains, so-called *bursts*, at 10 Hz, with each train consisting of 1–2700 pulses. The temporal length of the burst is defined by the duration of the accelerator radiofrequency (RF) pulses, with a maximum train duration of 0.6 ms (Fig. 2 ▸). As a consequence, 2700 pulses can be delivered by the machine at a pulse repetition rate of 4.5 MHz. Smaller intra-train repetition rates of *e.g.* 1.125 MHz or less can be achieved at the expense of the number of total pulses available in a single train; at 1.125 MHz, this amounts to 675 pulses, while at 376 kHz to only 225 pulses (see Fig. 2 ▸). In addition, these pulses have to be distributed among all three SASE beamlines, which could limit the available number of X-ray pulses for each experiment to 900 at 4.5 MHz. Details of the pulse distribution scheme are currently under investigation, and the facility aims to offer a most flexible electron bunch filling pattern for each of the three undulators. At present, 120 pulses per pulse train are routinely offered at the SASE1 instruments, each separated by 889 ns (or 1.125 MHz intra-train repetition rate); however, more pulses will become available over the course of next year, eventually reaching 2700 pulses at 4.5 MHz.

## 4. Overview of the FXE experimental hutch   

**Figure 3:**
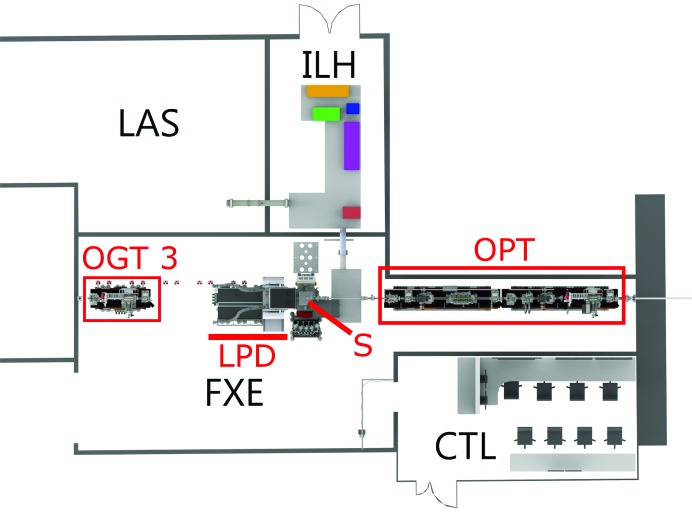
Top view of the scientific instrument FXE and its adjacent laboratories. The pump–probe laser system (LAS) delivers the beam into the instrument laser hutch (ILH), where it is further treated, guided into the experimental hutch (FXE), and focused onto the sample (S), where the X-rays (entering from the right into FXE) are guided through the optics branch (OPT) to probe the light-triggered processes. Behind the sample is the LPD detector and the post-sample diagnostics branch (OGT3). All X-ray experiments are remote-controlled from the FXE control room (CTL).

The experimental hutch of the scientific instrument FXE consists of three main parts: the 8 m-long optics branch (OPT in the right part of the FXE area in Fig. 3 ▸), the sample environment (S) in the center, including the robot arm mounted on a steel tower and two secondary XES spectrometers (central part of the FXE area in Fig. 3 ▸) as well as the LPD (Hart *et al.*, 2012 ▸), and the post-sample diagnostics branch (left part of the FXE area in Fig. 3 ▸). The FXE instrument is designed to permit simultaneous usage of the spectrometers near the sample position and the LPD behind it, in pump–probe geometry and dominantly under ambient conditions (helium atmosphere).

The instrument design seeks to maintain a flexible sample environment, with space available around the sample for desired modifications or additions. Therefore, the XES detectors are mounted on an inverted robot arm, hanging from a vibrationally stabilized robot tower. A motorized sample stack goniometer is placed 40 cm below the X-ray beam axis, allowing for the inclusion of additional equipment, *e.g.* a small goniometer for solid samples. A particular design aim is to facilitate liquid sample investigations, where the liquid flows through high-speed jets that can contain solvated chemicals or dissolved nanoparticles.

### 4.1. FXE optics branch   

**Figure 4:**
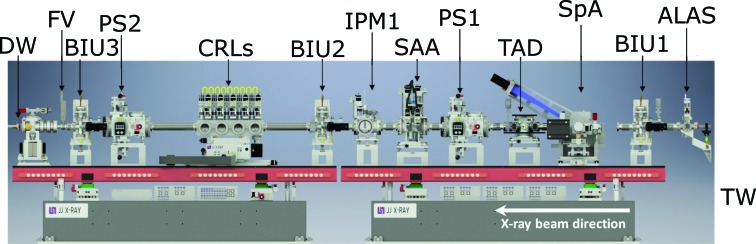
The upstream optics branch of FXE, with its various diagnosis and beam shaping components. Be-based CRLs focus the beam onto the sample 5 m downstream. The X-ray beam exits the UHV environment on the far left via a water-cooled diamond window (50 and 100 µm window thicknesses available). Legend: DW = diamond window, FV = fast valve, BIU = beam imaging unit, PS = power slit, CRL = beryllium lenses, IPM = intensity/position monitor, SAA = solid attenuator assembly, TAD = time arrival detector, SpA = single-shot spectrum analyzer, ALAS = alignment laser, TW = tunnel wall.

A Si(111) monochromator is installed 10 m upstream from the FXE photon beam tunnel transport wall (inside the tunnel). Its four-bounce geometry conserves the same X-ray beam axis for pink as well as for monochromatic beams. This is an important feature for pump–probe studies utilizing an external optical laser for sample excitation. Downstream from the monochromator, a retractable beam splitter (diamond grating) can send the main beam together with both −1st- and +1st-order diffracted beams into the optics branch (OPT in Fig. 3 ▸; also Fig. 4 ▸) of the FXE instrument. These diffracted beams are used for a curved crystal based single-shot spectrum analyzer (SpA) and a foil-based time arrival detector (TAD), respectively, to determine the spectrum of every single shot and the relative arrival time of each X-ray pulse with respect to the optical laser pulse, respectively. Moreover, the optical branch contains several diagnostics and beam shaping components on two optical granite tables: three beam imaging units (BIUs), two power slit systems (PS1 and PS2), each slit capable of withstanding the unattenuated X-ray beam, a solid attenuator assembly (SAA), and ten-element Be-lens focusing optics (CRLs).

The transmission of the entire beamline from source to end has been determined to be >70%, after optimization of the components in the tunnel, mainly three horizontally deflecting mirrors and the collimating Be-lens stack, and those in the optics branch.

In the following sections we will describe the characteristics and performance of a few components of the FXE instrument during the commissioning in 2018.

#### 4.1.1. Beam imaging units and single-shot intensity position monitor   

The FXE instrument is equipped with four identical BIUs, three in the optics branch before the sample and one in the post-sample diagnostics branch. They are designed for visual imaging of the spatial distribution of the incoming X-ray beam intensity using visible emission from either a Ce-doped YAG crystal (Ce:YAG) for single-bunch or low-intensity operation, or a thin polycrystalline diamond for burst-mode operation or when high transmission is desired. Each BIU is equipped with a motorized water-cooled copper rod supporting up to four different targets for beam visualization. Currently, both YAG and diamond targets have a thickness of 50 µm; the extra two could either be spares of the same YAG/diamond and different thickness or be made of different materials (*e.g.* BN, ceramic) suitable for higher energy or full burst mode at 4.5 MHz. The targets are tilted by 45° with respect to the X-ray beam propagation direction, and the visible emission is collected by a CCD camera parallel to the targets under 90° to the X-ray beam, synchronized to the 10 Hz pulse train. In this configuration the BIUs have a resolution of approximately 25 µm pixel^−1^, which is sufficient for good diagnostics of the unfocused beam (or slightly focused in the case of BIU3).

**Figure 5:**
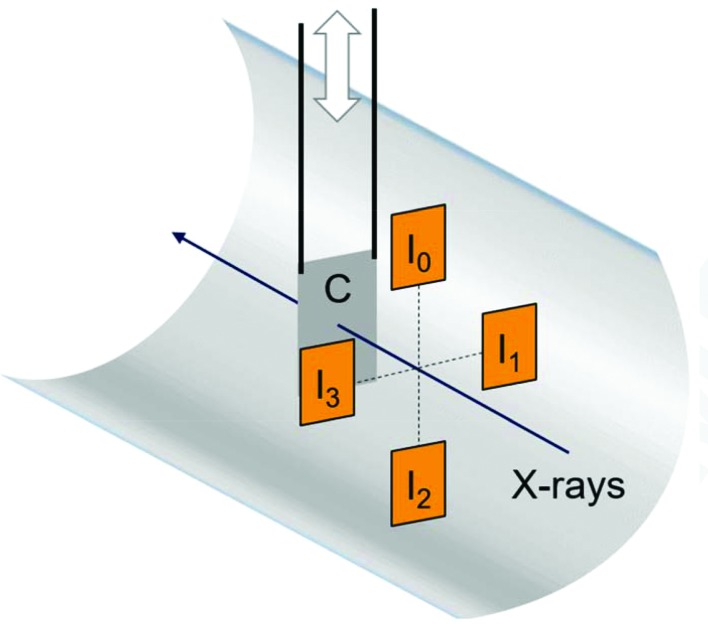
Schematic geometry of the IPM in the FXE instrument. Four APDs (orange, labeled I_0_, I_1_, I_2_ and I_3_) collect backscattered (and fluorescence) photons from a 15 µm-thick screen (C) made of nanocrystalline diamond with grain size specified <50 nm. The diodes are mounted on a linear translation stage, which permits the movement of the entire diode assembly closer to or further away from the backscattering foil, depending on the actual incident flux and photon energy.

**Figure 6:**
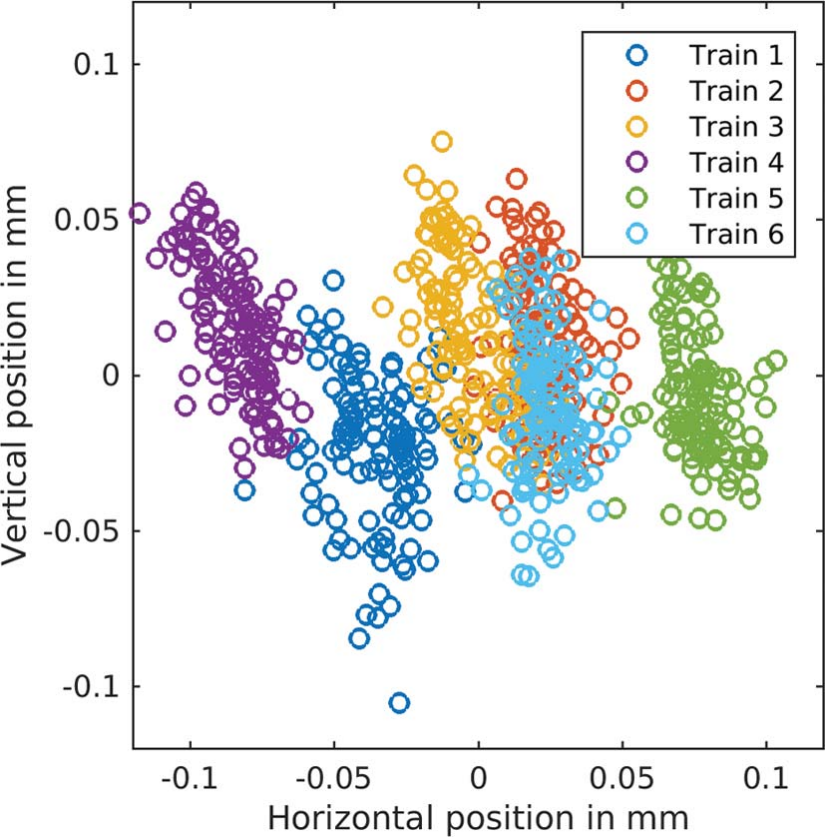
Single-pulse-resolved X-ray arrival position at the FXE instrument for 10 Hz consecutive trains (color coded) arriving at a 1.1 MHz intra-train repetition rate. One observes a fairly random jitter of the centroid horizontal beam position from train to train, whereas intra-train jitter occurs predominantly in the vertical plane, judging by absolute amplitudes. Considering that this measurement was performed on the collimated beam with FWHM intensity of 700 µm (vertical) and 230 µm (horizontal), the jitter amplitudes relative to the beam size are dominated by the horizontal contribution.

Two intensity and position monitors (IPMs), one schematically shown in Fig. 5 ▸, are included upstream and downstream of the sample position to enable reproducible X-ray beam propagation through the FXE instrument. Both IPMs are equally equipped with retractable diamond screens and four silicon diodes picking up scattered X-rays (dominated by Compton scattering) as well as fluorescence photons, since the avalanche photodiodes (APDs) operate without optical shielding. Because of the arrangement of diodes in quadrants, the horizontal as well as vertical beam position can be determined from the difference signal, while the total signal is proportional to the pulse energy. The dynamic range is extended (by about a factor of ten) by a linear translation stage, which enables different screen–detector distances. This addition is different to the otherwise similar design at other FEL facilities (Feng *et al.*, 2011 ▸; Chollet *et al.*, 2015 ▸). The screens are made of 15 µm-thick nanocrystalline diamond (grain size < 50 nm). The choice of detectors includes either APDs or PIN diodes, Hamamatsu S8664-1010 or S3590-09, respectively, with 10 × 10 mm sensor size and mounted in backscattering geometry. The resulting electronic signal is capacitively coupled into a fast 12-bit analog-to-digital converter (ADC) (SP devices ADQ412). Commonly, bias voltages of 200 V are applied to the APDs, and a signal with sub-200 ns FWHM temporal response is measured, enabling the read-out of single X-ray pulse energies up to more than 1.1 MHz repetition rates. The integrated signals from each APD allow calculation of the beam position from the normalized difference of opposite APDs, while their sum is proportional to the pulse energy. The spatial information is calibrated by comparison with the beam position on a BIU, while deliberately sweeping the beam over the active area. The resolution of a similar device was shown to be better than 5 µm by moving the diodes inside the IPM on an absolute scale (Feng *et al.*, 2011 ▸). Fig. 6 ▸ shows experimental results from the device, monitoring the horizontal position of each X-ray pulse (9.3 keV) in consecutive trains at 1.1 MHz. Horizontal inter-train pointing jitter amounts to 130 µm (FWHM), whereas intra-train jitter is only 27 µm, for a horizontal beam size of 230 µm (FWHM). In the vertical plane (data not shown), the inter- and intra-train jitter have equal amplitudes of 50 µm with a vertical beam size of 700 µm. This result confirms the overall superior intra-train beam stability of 12% (horizontal) and 7% (vertical) of the respective beam sizes.

The information from the IPM is also used for normalization of detector data and as a feedback signal acting on the pitch of the last deflecting X-ray mirror (M3) in the photon tunnel in order to compensate for slower drifts of the X-ray beam on the scale of seconds to hours.

#### 4.1.2. Beam-splitting diamond grating   

A retractable beam splitter in the form of a grooved diamond grating is installed 8 m upstream of the first component in the FXE optics branch, thus inside and near the end of the SASE1 tunnel section. The unit can support up to four gratings, which can be exchanged according to the experiment needs including diffraction efficiency, beam separation and X-ray energy. The gratings are made of diamond with 10 µm thickness and are supported by a silicon frame. Currently, we have three diamond gratings installed, one with 150 nm pitch and two with 200 nm pitch, that were manufactured by the group of C. David from the X-ray Optics and Applications Group in the Laboratory for Micro- and Nanotechnology of the Paul Scherrer Institut in Switzerland.

**Figure 7:**
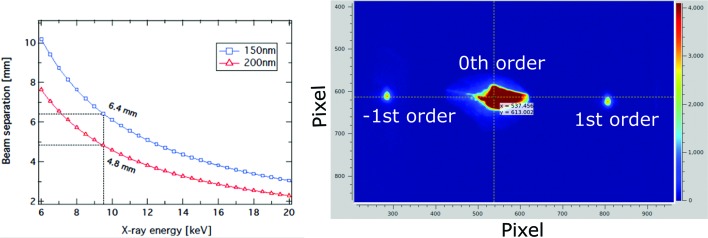
(Left) Calculated separation between the main beam and the +1st and −1st diffracted beams at the position of BIU1 as a function of the X-ray energy for the two different grating pitches currently installed at FXE. Right: image of the 0th, 1st and −1st X-ray beam diffracted by one of the 150 nm-pitch diamond gratings as seen by BIU1.

In Fig. 7 ▸ (left), we show the expected beam separation between the main beam and the +1st and −1st diffracted beams in the operational energy range of FXE, at the position of BIU1 on the FXE optics branch. On the right side, an image of the 0th, 1st and −1st order of the 9.3 keV X-ray beam diffracted by one of the 150 nm-pitch diamond gratings as detected by BIU1 is shown. The separation between the 0th and the +/−1st order is around 270 pixels.

With a conversion factor of roughly 25 µm/pixel, this corresponds to an estimated separation of around 6.7 mm at the position of BIU1. The beam splitter can serve different diagnostic components, such as the SpA (see Section 7.1) or the TAD (Section 7.2), and is also used at other FEL facilities (Rich *et al.*, 2016 ▸; Makita *et al.*, 2015 ▸; Katayama *et al.*, 2016 ▸; Rehanek *et al.*, 2017 ▸).

#### 4.1.3. Compound refractive lenses for beam focusing   

**Figure 8:**
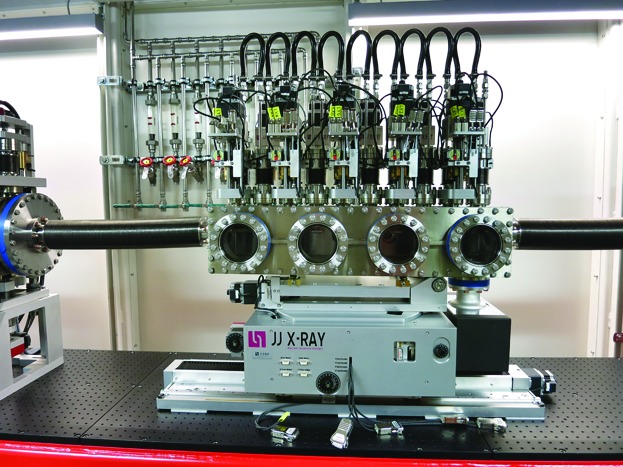
The ten-axis Be-lens chamber allows one to focus all X-ray energies onto the sample 5 m downstream. A chamber translation stage further adjusts the spot sizes within its stroke of ±50 cm.

The lens stack of FXE (Fig. 8 ▸) comprises 70 beryllium lenses with 1.5 and 3 mm apertures, distributed on ten arms with up to ten lenses on each arm.

This arrangement permits us to focus all X-ray energies from 5 to 20 keV onto the sample, which is located 5 m downstream, when we also use the 50 cm translation stroke of the entire Be-lens chamber. The effective aperture (Lengeler *et al.*, 1999 ▸) defined by the geometry of the lens and its wavelength-dependent X-ray absorption is around 1 mm over the 5–20 keV wavelength range. The performance of this device, in terms of focusing capabilities, is discussed in more detail in Section 6 (Fig. 14).

### 4.2. Sample interaction area   

**Figure 9:**
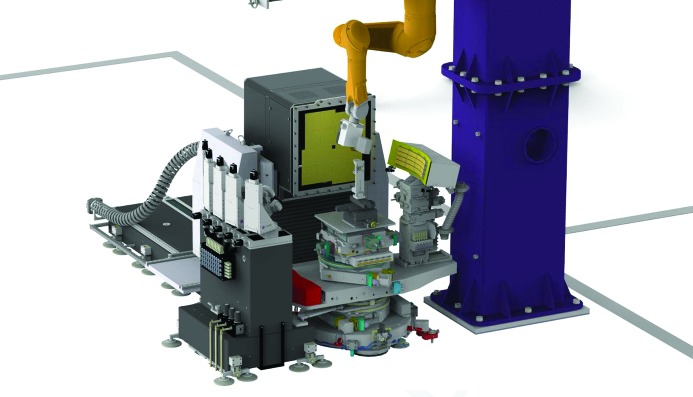
Illustration of the sample interaction area at FXE with a setup for liquid samples in the X-ray focus. Two X-ray spectrometers, von Hamos (right) and Johann (left), and the LPD (used for forward-scattering/diffraction experiments) surround the sample. A robot (orange) holds the 2D MHz-compatible strip detector to detect XES from the 16-element von Hamos type X-ray emission spectrometer. The sample environment design enables free space around the actual sample, and this opens up room for flexible modifications, as demanded by different groups of experiments.

The FXE instrument is very flexible in terms of sample environment. The sample interaction point is centered on top of a ten-axis sample stage from Huber (gray component in the center of Fig. 9 ▸) and a tower (blue, right in Fig. 9 ▸) that supports a robot arm (Stäubli, orange in Fig. 9 ▸) holding several different detectors. These detectors are mainly for secondary spectroscopy purposes in contrast to LCLS, which uses this configuration mainly for forward-scattering detectors (Chollet *et al.*, 2015 ▸). Connected to the sample-mounting stack (SMS) are two high-energy-resolution X-ray spectrometers, one operating in dispersive mode (von Hamos, right in Fig. 9 ▸) and the other in scanning mode (Johann, left in Fig. 9 ▸). The spectrometers can be rotated around the sample, allowing for spectroscopic experiments in different geometries.

Further downstream, the LPD can be placed as close as 60 mm from the sample interaction point, which enables collection of scattering up to about 65° without shadowing. Alternatively, the LPD can be placed as far as about 7 m from the sample interaction point. The LPD is mounted on a granite block equipped with three independent motorized stages; the translation along the beam direction has a 1.5 m range. The whole LPD mount moves via air pads and can be easily aligned due to the presence of docking stations along the beam propagation direction.

#### 4.2.1. Liquid jet sample environment   

**Figure 10:**
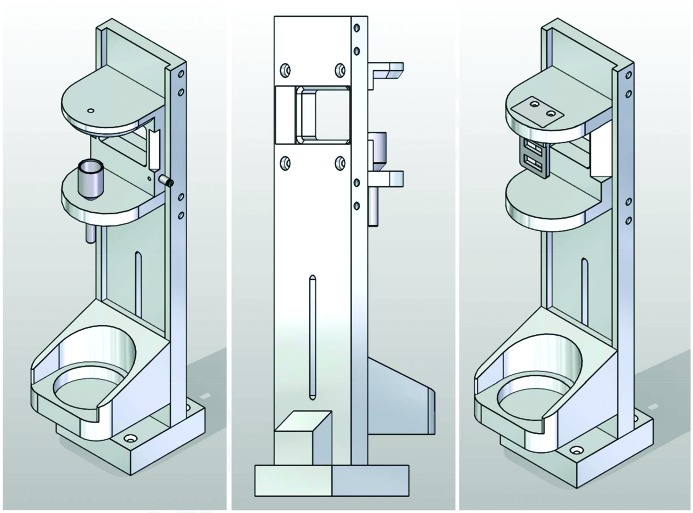
(Left, center) Universal support for liquid jets, including a catcher and bottle holder with variable height adjustment. (Right) On the same universal support, several beam characterization/diagnostic mounts can be placed, here a support for beam imaging, holding 50 µm large area Ce:YAG and diamond screens.

Experiments with liquid samples greatly benefit from the capabilities provided by FXE and the high repetition rate of the EuXFEL. In order to provide a flexible environment for liquid samples, a universal support was designed which is capable of holding several different jets, as well as beam characterization and diagnostics (YAG or diamond screens, timing diodes, pinholes, laser beam profiler *etc*.), at the sample position. This support is shown schematically in Fig. 10 ▸. It is designed such that any jet mount or alternatively beam characterization/diagnostics is placed at the same distance from the support edge, maintaining the beam–sample interaction point to within 10 µm. This facilitates the fast exchange of jets and diagnostics during experiments, while keeping spatial overlap of laser and X-rays, which is of fundamental importance in pump–probe experiments. The support for liquid jets was designed to have a wide opening of almost 180° downstream from the sample, allowing the concomitant use of both X-ray spectrometers and forward-direction scattering without shadowing any of them. The whole support can be covered with thin polymer foils (*e.g.* Kapton, Mylar), and a gas inlet with fast connector (Festo) can be used to create an inert or protective atmosphere around the liquid jet. On top of the SMS, three independent orthogonal translation stages can be used to further align the liquid jet sample environment onto the X-ray beam.

### 4.3. Forward scattering with the Large Pixel Detector   

**Figure 11:**
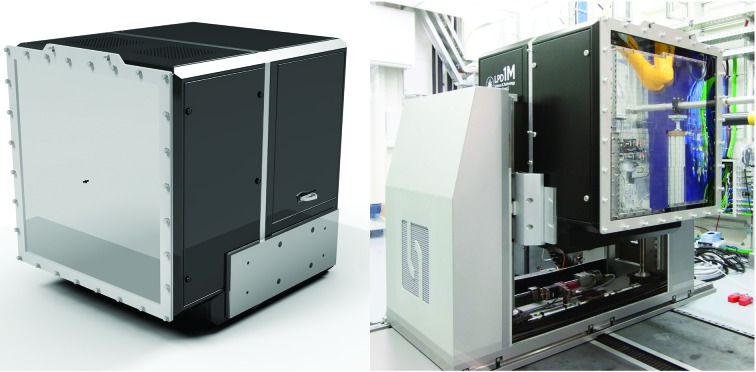
The Large Pixel Detector (left: design, right: as installed inside the FXE hutch) has 1 million pixels, each with 0.5 × 0.5 mm size. The detector can store at maximum 510 images sampled with a frequency of 4.5 MHz. Each single-shot image has a dynamic range of about 1 × 10^5^ 12 keV photons.

The high photon energy, high brilliance and high repetition rate capabilities of the EuXFEL machine set demanding requirements for the main scattering and diffraction detector. The LPD (Fig. 11 ▸) was designed, developed and initially commissioned at the Rutherford Appleton Laboratory, Science and Technology Facilities Council, in Oxford, UK, in collaboration with EuXFEL, strictly following the requirements of the FXE instrument design. The LPD is a hybrid pixel detector with square pixels of 500 µm × 500 µm in size. Each pixel has 512 analog memory cells allowing the storage of up to 510 images recorded with 100 ns integration time at the maximum EuXFEL frame rate of 4.5 MHz. It then transfers these images within 99.6 ms to a data acquisition system, thus operating in a burst mode compatible with the X-ray pulse pattern of the EuXFEL. The LPD provides a dynamic range of 10^5^ photons at 12 keV enabled by the three parallel gain stages with three gain factors of 1×, 10× or 100× and two preamplifier settings with a 50 pF or 5 pF feedback capacity. The latter provides sub-single-photon noise at 14 keV at the expense of dynamic range.

**Figure 12:**
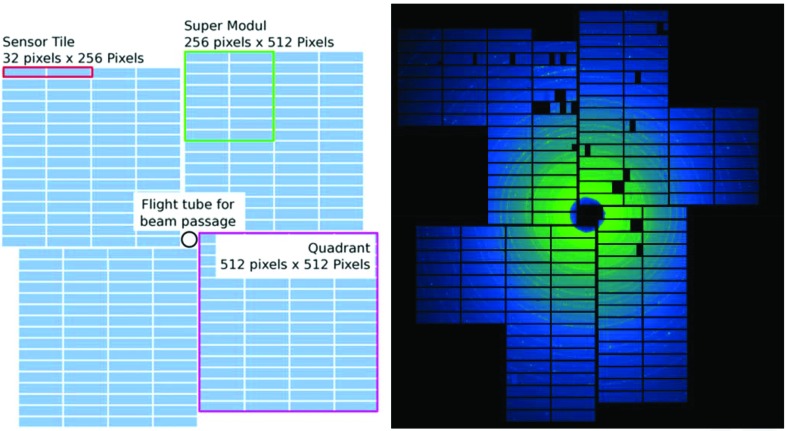
(Left) Schematic view of the LPD sensor plane showing the arrangement of sensor tiles. The quadrants can be moved in an iris in a clockwise and counter-clockwise direction to open and close the central beam hole. (Right) Static X-ray diffraction pattern of a calibration powder sample (NIST LaB_6_) measured at 14 keV incident X-ray energy. Note that at the time of this experiment the LPD detector was not fully equipped with all supermodules.

The most appropriate gain factor for each pixel in each image is automatically selected after signal amplification and before data are transferred from the analog memory to the data acquisition system. For photon energies >18 keV, the detector provides single-photon sensitivity at the 3σ level. The 500 µm-thick Si sensor allows for X-ray detection with a quantum efficiency >90% in the 5–13 keV photon energy range and approximately 30% at 20 keV. The sensitive area consists of 1024 × 1024 pixels in total, grouped in 256 exchangeable rectangular tiles (128 × 32 pixels, Fig. 12 ▸).

These tiles are arranged into mostly independent supermodules consisting of 16 tiles each. Furthermore, the supermodules are grouped into four quadrants that can be moved in an iris-like pattern in the detection plane to form an adjustable square central hole for the direct X-ray beam to pass through to avoid radiation and physical damage. A ‘veto’ capability can reduce the effective detection frame rate based on a configurable ‘veto’ pattern, matching, for example, a sparser pulse structure, or allow one to discard poor images based on an external veto signal. Real-time diagnostic devices can, for example, provide such an external veto signal to improve the overall number of good quality images transmitted to the data acquisition system. In particular, when the total number of pulses per burst exceeds the 510 available memory cells, the veto mechanism allows detection and storage of a maximum number of good images. Raw images are corrected on a per-pixel, per-memory-cell and per-gain basis for offset and relative gain. A geometric correction of the relative pixel positions based on photographic metrology data is available. GPU-based corrections have been implemented for online feedback, and can handle image rates of up to 256 Mpixel images per second (Hauf *et al.*, 2019 ▸; Fangohr *et al.*, 2018 ▸). The respective calibration parameters were established after delivery of the LPD to EuXFEL and are stored in the calibration database; corrections from earlier set points can be reapplied to the raw data upon request and are resolved by detector operating conditions (Kuster *et al.*, 2014 ▸). A full set of calibration parameters for LPD consists of billions of parameters, totaling approximately 40 GByte in size.

### 4.4. X-ray emission spectroscopy with an energy-dispersive spectrometer   

FXE was designed to be equipped with two independent high-energy-resolution X-ray spectrometers, one operating in dispersive mode in a von Hamos geometry (von Hámos, 1932 ▸) and the other in scanning mode with curved crystals in Johann geometry (Bergmann & Cramer, 1998 ▸). Both X-ray spectrometers are based on designs already implemented at many synchrotrons and other FELs (Szlachetko *et al.*, 2012 ▸; Alonso-Mori *et al.*, 2012 ▸, 2015 ▸; Britz *et al.*, 2016 ▸). Here, we describe the dispersive-type spectrometer, which is based on the von Hamos geometry.

**Figure 13:**
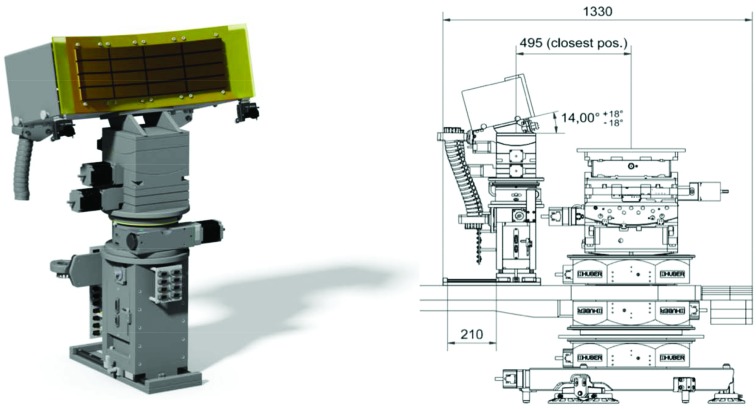
A 3D model of the FXE von Hamos spectrometer. It can hold up to 16 cylindrically bent crystal analyzers with 500 mm radius of curvature allowing for motorized alignment of each of them. The spectrometer is fixed to the sample mounting stack, which consists of various motorized stages, including a rotation stage that allows one to position the spectrometer at different scattering angles with respect to the sample.

The von Hamos spectrometer (VHS) at FXE has a multi-crystal design, containing up to 16 cylindrically bent curved analyzer crystals with 500 mm radius (Fig. 13 ▸). Each crystal can be individually rotated with very high resolution around a horizontal and a vertical rotation axis in its diffraction plane by means of three stepper motors arranged in a triangular pattern. Each crystal can easily be removed and re-installed by a magnetic coupling, allowing for very fast and reproducible changes in the analyzer configuration. The VHS spectrometer is attached to the SMS and has ten degrees of freedom: three motorized orthogonal rotations and translations, and a manual translation (Fig. 9 ▸). The entire VHS system can be rotated around the SMS vertical axis by a large rotation stage, allowing spectroscopic experiments in different geometries. The complete VHS can be operated in air; or, alternatively, a helium atmosphere enclosed in a flexible container with thin windows transparent to X-rays can be used to minimize attenuation by the air path sample VHS detector. The operation and performance of von Hamos spectrometers regarding energy resolution and signal quality, including on FEL time-resolved experiments, have already been covered in several publications and will not be described here (Szlachetko *et al.*, 2012 ▸, 2017 ▸; Alonso-Mori *et al.*, 2012 ▸; Britz *et al.*, 2016 ▸).

## 5. FXE optical laser systems   

Several pulsed optical laser beams synchronized to the burst-mode sequence of X-ray pulses are available for experiments. The lasers are located in different rooms in close vicinity to the X-ray hutch (Fig. 3 ▸) in order to keep laser path lengths reasonably short as well as to permit work on these systems even when X-rays in the experiment hutch prohibit access (to that room).

While the EuXFEL laser system is situated in the larger laser hutch (LAS in Fig. 3 ▸), the commercial Tangerine laser is located in the FXE instrument laser hutch (ILH in Fig. 3 ▸). This allows users to choose between different beams with different properties (wavelength, pulse duration). The currently tested wavelengths are summarized in Table 1 ▸. More conversion components (*e.g.* *TOPAS*) will be installed soon, which will further increase the available wavelengths, with the goal of eventually serving the entire 200–2000 nm range. THz radiation is also on the upgrade program and will add to the available portfolio of laser wavelengths.

## 6. Performance status of FXE   

The FXE instrument has served early users since September 2017, while constantly improving its conditions and components for time-resolved investigations with fs resolution. In this section, we demonstrate the current status of this scientific instrument, by showing key properties and measurements. Time-resolved studies require unique measurement conditions in order to achieve high-quality data with fs resolution. Specific experimental parameters, instrumentation and procedures that play a key role are the spot sizes of the laser and X-ray pulses, their stability, achieving spatial and temporal overlap of both pules at the sample position, the sample environment itself, online analysis tools, and the measurement protocol, including possible reference samples.

**Figure 14:**
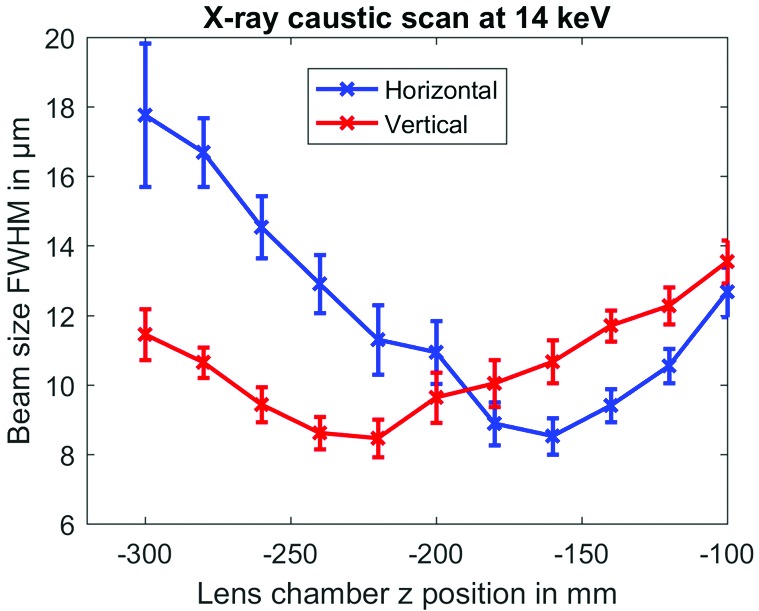
A caustic scan at the sample position demonstrating the focal properties at 14 keV. The data were obtained by moving the lens chamber in the beam direction and recording the fluorescence from a 50 µm-thin YAG screen. Error bars denote a 1σ standard deviation of the X-ray single-pulse beam size.

As the spot size of the X-ray beam in laser pump–X-ray probe measurements needs to be smaller than the laser spot size in order to probe a uniformly excited sample volume, a first requirement is a tunable, small X-ray focus. At FXE, the X-ray beam from the source is collimated with a set of Be lenses 215 m from the source (Fig. 1 ▸) and focused with another set of Be lenses (located 5 m upstream from the sample) onto the sample (Fig. 8 ▸). Caustic scans around the sample position yield about 10 µm focal spot sizes in both horizontal and vertical directions (Fig. 14 ▸, here for 14 keV radiation). Typically, the laser is then focused to a slightly bigger spot size on the sample, around 50–150 µm. Given these focal sizes for different target systems, different strategies are pursued in order to ensure that each, or a specific number of, pump–probe shots illuminates a fresh sample.

**Figure 15:**
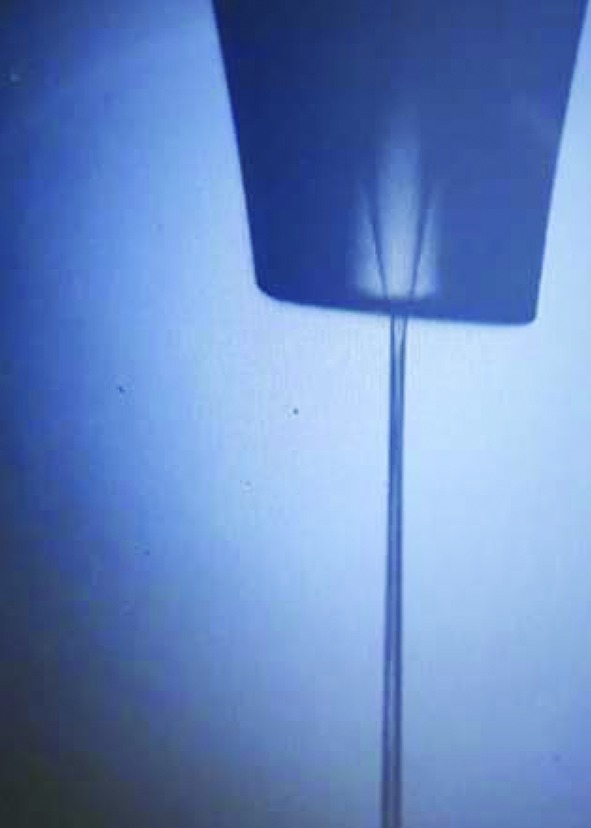
Photograph of a 100 µm-diameter round liquid jet with quartz glass capillary at a flow speed of 30 m s^−1^.

At FXE, liquids can be supplied by micro-jet systems. The fastest jet flow speeds can be reached with round jets (Fig. 15 ▸), and, currently for the routinely used 100 µm-diameter nozzles, flow speeds of about 30 m s^−1^ can be achieved with a laminar flow. Thus, a combination of a 50 µm laser spot and a 564 kHz repetition rate of the FEL will guarantee that every pump–probe shot will interact with a fresh sample volume. For solid-state samples, a sample scanning procedure is implemented; the solid-state target is subject to a raster scan with each spot on the target exposed to a limited number of pump–probe exposures. To illuminate a fresh spot on the solid-state target, step sizes for the raster scan have to be sufficiently large. For successful sample motion to X-ray arrival synchronization, an X-ray pulse train on demand functionality is used that only allows the illumination of a sample after it has reached its designated position.

Another important parameter is the spatial stability of both the X-ray and laser beams at the sample position. A detailed discussion of the X-ray beam stability at FXE is given in Section 4.1.1 for the IPM. Information on the beam position from the BIUs or the IPM is automatically fed back to the piezo motors of M3, correcting spatial drifts on timescales longer than 2 s. The stability of the optical laser beams will be further controlled by temperature-stabilizing beam path enclosures. The strategy to achieve the spatial overlap between the X-ray and laser pulses is based on first imaging the relative positions of both beams on a YAG screen at the sample position. Subsequently the laser beam is adjusted to the X-ray position on the YAG by a stepper-motor-controlled mirror in the laser beam path. The coarse temporal overlap (∼100 ps) between the X-ray and laser pulses is achieved by recording the temporal signature of both pulses at the sample position using a fast photodiode, Hamamatsu GE 4176, with a rise time of *circa* 30 ps. A Tektronix oscilloscope is used to monitor the relative timing of the pulses. Up to now, the pump–probe laser is synchronized via a RF setup. RF frequencies of the accelerator’s master oscillator serve as reference signals. The phase of the optical pump laser is then shifted to adjust the relative timing between the laser and the X-ray pulses. A more accurate determination of time-zero, currently around 300 fs, is done on the signal from the sample or a reference sample. In this case, the time-resolved XES signal is used as a figure of merit for both temporal and spatial overlap optimizations. For fast and reliable alignment, well-known calibration samples can be used to pre-adjust temporal and spatial overlap before introducing the user sample.

**Figure 16:**
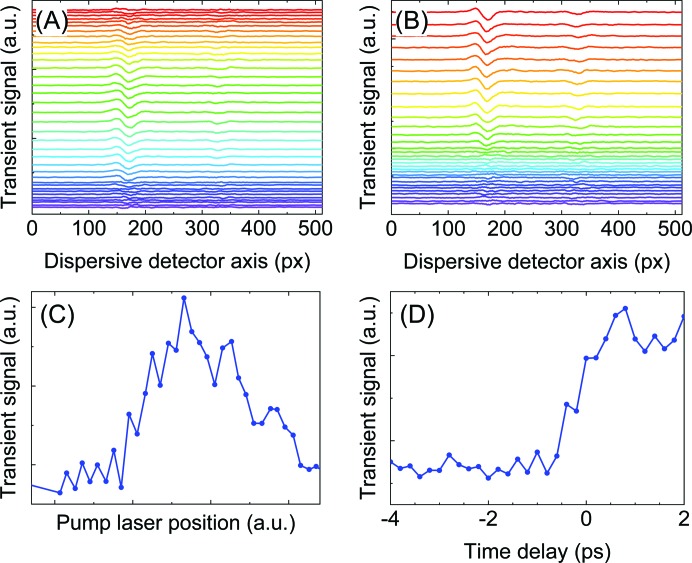
(*a*), (*b*) Pump–probe XES signal of 15 m*M* solution of Fe(bpy)_3_^2+^ in aceto­nitrile while scanning the vertical position of the last mirror in the optical laser beam path (color coded in the upper picture). The maximum of the transient signal marks optimal spatial overlap of the laser and the X-ray beams. (*c*), (*d*) Then, the phase shifter of the optical laser is scanned to accurately determine time-zero (Fig. 18 ▸, right). The phase shifter value is color coded in the upper picture. Images were recorded with a CCD (from GreatEyes Co.) with 10 Hz frame rate. Exposure time was set to 5 s for every data point in (*c*) and (*d*), scans were done twice, once with exposure of the sample by the pump laser, and once without. The transient signals were obtained as the difference in sums of all background-corrected laser-on- and laser-off spectra recorded with the von Hamos spectrometer.

This alignment procedure is illustrated in Fig. 16 ▸ for the case of a 15 m*M* solution of Fe^II^(bpy)_3_^2+^ (bpy = bipyridine) in aceto­nitrile. Once the static emission signal from the reference sample is found, the horizontal and vertical positions of the last mirror in the optical laser beam path are scanned while the transient pump–probe XES signal is recorded, as illustrated in Figs. 16 ▸(*a*)–16 ▸(*b*). The maximum of the transient signal marks optimal spatial overlap of the laser and the X-ray beams. The phase shifter of the optical laser is then scanned to accurately determine time-zero as shown in Figs. 16 ▸(*c*)–16 ▸(*d*).

## 7. Upcoming FXE components   

In the FXE optics branch, there are still components in the early stages of commissioning, which we describe below.

### 7.1. Spectrum analyzer   

**Figure 17:**
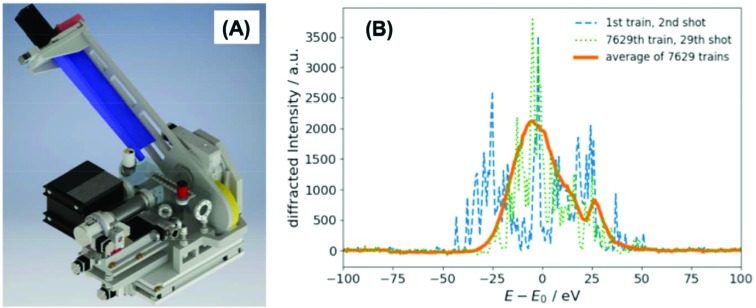
(*a*) 3D model of the spectrum analyzer allowing for mounting of four bent crystals. (*b*) Two arbitrarily selected single-shot SASE spectra (and the averaged spectrum after 12 min collection time) using a curved Ge(220) crystal at the incident X-ray photon energy of 9.3 keV.

The SpA will resolve the spectral distribution of the pink beam. Its operating principle is based on a dispersive diffraction setup (Zhu *et al.*, 2012 ▸; Makita *et al.*, 2015 ▸; Rich *et al.*, 2016 ▸; Rehanek *et al.*, 2017 ▸; Boesenberg *et al.*, 2017 ▸). The SpA consists of curved, thin, single crystals of Si(111), Si(110), Ge(110) (100 µm) or diamond/CVD foil and a 1D strip detector (Gotthard) as illustrated in Fig. 17 ▸(*a*). The crystals are mounted on a temperature-stabilized copper holder with five degrees of freedom: movement in and out of the X-ray beam, to the center of the X-ray beam, and to the center of the detector rotation as well as tilting and Bragg-angle rotation. The convex bent crystals spectrally disperse the first-order diffracted X-ray beam from the upstream diamond grating onto the detector. The size of the impinging X-ray beam and the curvature of the crystal define the theoretical recordable energy window. In conjunction with a second SpA downstream of the sample (also to be commissioned), it will be possible to carry out self-normalized X-ray absorption measurements.

Note that, given the high X-ray transmission, the SpA can be operated in parallel with actual measurements at the sample position. The device is capable of recording a shot-to-shot dispersed incident SASE spectrum at 564 kHz at the moment, where the repetition rate limit is imposed by the Gotthard detector read-out speed (limited to 0.8 MHz). The example of a SASE spectrum at the nominal central wavelength of *E*_0_ = 9.3 keV dispersed onto the Gotthard detector using a Ge(220) bent crystal is shown in Fig. 17 ▸(*b*).

### 7.2. Timing tool   

**Figure 18:**
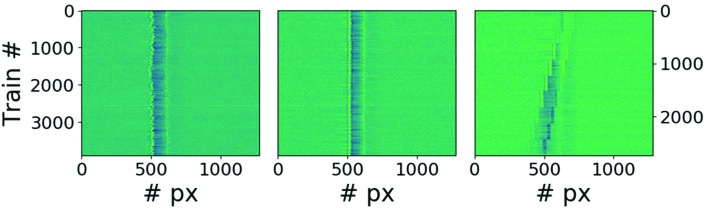
(Left) Raw data of radiofrequency-synchronized laser jitter measurements. (Middle) Raw data of an optically synchronized laser jitter measurement. (Right) A time calibration measurement using an optically synchronized laser with 500 fs delay steps.

The TAD measures the relative time delay (or time arrival) between the synchronized optical laser and X-ray FEL pulses. This measurement has to be performed for each incident optical laser and X-ray pulse to allow jitter-corrected shot-to-shot pump–probe experiments. In principle, three methods can be employed: spatial, spectral and interferometric encoding. An extensive review of each of these techniques and a conceptual design of temporal diagnostics devices that are potentially feasible at FXE can be found in the work of Harmand *et al.* (2013 ▸), Hartmann *et al.* (2014 ▸), Schulz *et al.* (2015 ▸) and Katayama *et al.* (2016 ▸). The mechanical design of the TAD chamber at FXE is described in detail in the Technical Design Report for FXE (Bressler *et al.*, 2012 ▸). Early commissioning measurements are presented in Fig. 18 ▸. For all subfigures the vertical axis is merely showing subsequent trains of pulses at shot 50, while the *x* axis is simply the dispersive axis of a spectrometer used to capture the time arrival signal. A detailed analysis and thus implementation into the data stream of FXE is ongoing.

### 7.3. Solid-state sample environment   

**Figure 19:**
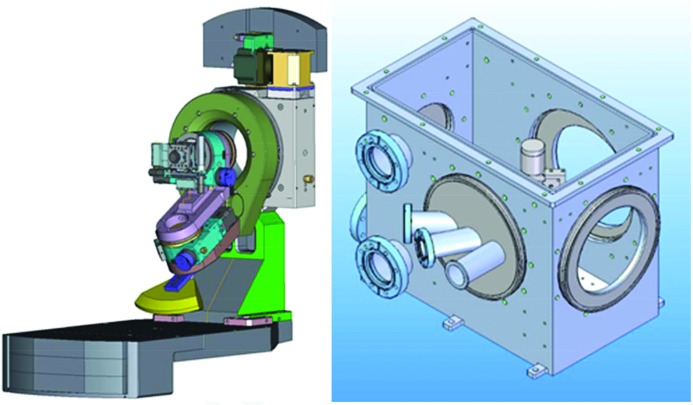
(Left) Kappa goniometer at the FXE instrument. For specific FEL applications, samples can be translated by 50 × 50 mm during the measurement. (Right) Versatile vacuum chamber at the FXE instrument. X-rays and the optical laser enter through the front-left flange, and the front-right and rear-right flange are used for emission spectroscopy and scattering, respectively.

Experiments on solid-state samples can be performed at FXE in air as well as in vacuum. A Kappa goniometer is the common sample stage for in-air experiments, such as grazing-incidence diffraction. It is shown schematically in Fig. 19 ▸ and is commonly installed onto the SMS (Fig. 9 ▸). Besides the choice of arbitrary incidence angles ω, φ and χ, it enables sample translation over a 50 × 50 mm range to avoid sample damage issues. During translation, the sample surface must stay in the interaction volume of pump and probe beams. In particular, in grazing-incidence experiments close to the total reflection angle, spatial inhomogeneities in sample height must be compensated with micrometre accuracies. This is achieved by optical distance measurements and a mechanical compensation strategy. In the case of oxygen-sensitive samples, He purging is employed.

A small and versatile vacuum chamber is available at FXE, which can be used for XSS, XRD, XES and XAS measurements. It is shown schematically in Fig. 19 ▸, where an off-center geometry is depicted, which is ideal for scattering and simultaneous emission spectroscopy experiments. Polycrystalline or amorphous samples can be mounted on fast translation axes to enable sample scanning in transmission geometry. Instead of the sample scanner, a helium cryostat can be installed in the chamber to enable experiments at temperatures between 4 and 400 K. The optical pump laser is injected into the vacuum either through an angled viewport (at roughly 25° to the X-rays or through the main beam-pipe, at an angle of 4° to the X-rays). Up to six motorized stages can be installed in the chamber; the default configuration is two translation stages for sample frames and two translation stages for the beamstop. Options for an in-vacuum goniometer are under investigation, but specific solutions can be realized by combination of commercial rotation and translation stages operated with stepper motors.

### 7.4. X-ray emission spectroscopy: Johann scanning spectrometer   

**Figure 20:**
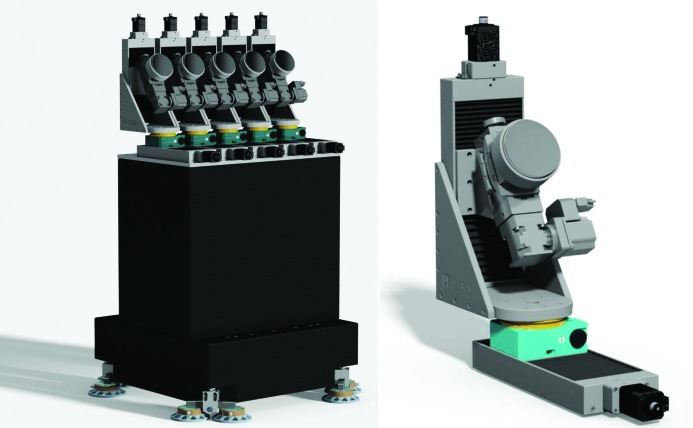
(Left) A 3D model of the five-crystal analyzer scanning XES spectrometer using the Johann geometry (JSS) with 1 m Rowland circle. (Right) The mechanical design of a single analyzer mount with four independent degrees of freedom is shown. The mount can hold 100 cm-diameter round analyzer crystals with various radii.

Next to the dispersive (von Hamos) XES spectrometer already described (see Section 4.4), the FXE instrument is also equipped with another point-to-point scanning X-ray spectrometer based on the Johann geometry. These types of devices have been successfully used in the synchrotron/FEL community, and most utilize the arrangement employing multiple bent crystal analyzers pioneered by Bergmann & Cramer (1998 ▸). The Johann scanning spectrometer (JSS) at FXE is equipped with five spherical analyzer crystals (Si and Ge) that can be independently moved with four degrees of freedom for fine alignment and during the energy scans (see Fig. 20 ▸). The typical analyzer radius is 1 m; however, the design permits the use of crystals with variable radii between 1.0 and 2.0 m, the former increasing the solid-angle coverage and the latter favoring a higher resolution according to the experimental needs. Similar to the dispersive spectrometer, the JSS is directly connected to the SMS and can be rotated along the sample vertical axis using air pads. Additionally, the air pads permit one to move the entire granite block holding the JSS and, in particular, away from the sample interaction region for those experiments requiring more available space near the sample position.

### 7.5. Avoiding collisions for the six-axis robot arm   

The six-axis robot is needed to align the X-ray emission detectors to the correct emission energy. This requires – especially for the Johann spectrometer – a combined movement of both the analyzing crystals and the X-ray emission detector during a given X-ray emission scan. This poses stringent conditions on the space the robot can occupy without damaging any ancillary equipment (*e.g.* the sample mount, the LPD forward-scattering detector *etc*). Additional safety measures must be put in place if the robot is going to be used via remote control, *e.g.* scanning positions during an experiment. These constraints require a low-level equipment protection system to be in place that can sense and stop the robot immediately if collision is going to occur. Unfortunately, such a low-level equipment protection is not practical on its own, as it can block the instrument during a scan without any corrective measure. Therefore, we need an additional special tool that checks the feasibility of movements beforehand and enables the execution of the permitted movements during the desired experimental scan. Hence, the complete trajectory of the required motions is verified beforehand against collision. This can be implemented in Karabo control software (Hauf *et al.*, 2019 ▸; Heisen *et al.*, 2013 ▸) by connecting to a Virtual Beamline Simulator which checks for collisions in 3D (du Boulay *et al.*, 2008 ▸; Atkinson *et al.*, 2009 ▸). Note that not only can physical collisions be prevented this way, but blockage of the detector view (*e.g.* shadowing of the effective scattering cone) can also be avoided. For a proper use, the instrumentation including the robot and its axes must be properly calibrated and positioned in the virtual reality (Brockhauser *et al.*, 2011 ▸; White *et al.*, 2018 ▸), which will happen during the next commissioning phase.

## 8. Conclusions   

The scientific instrument FXE is designed to permit a range of versatile experiments with optical pump and X-ray FEL radiation probe pulses, aiming to explore structural and electronic dynamics with fs time resolution. All equipment is now in place and pink beam experiments have been carried out since its inauguration in September 2017. Commissioning results show that we can perform experiments with 300 fs time resolution, which should improve following full implementation of the time arrival detector that will measure the relative arrival time between both pulses to better than 50 fs. Using the Be-lens optics, X-ray focal spot sizes of about 10 µm can be achieved routinely and can be conveniently overfilled with the optical laser pump pulses. The remaining equipment described in Section 5 is currently under commissioning, and FXE should become fully operational in 2019.

## Figures and Tables

**Table 1:** Available optical laser systems

Laser system	Fundamental wavelength (nm)	Fundamental pulse width (fs)	Fundamental pulse energy (µJ)	SHG	THG	FHG
Tangerine	1030	300	30	515 nm	353 nm	257 nm†
				300 fs	250 fs	
				10 µJ	0.7 µJ	
PP1	800	15‡	250	400 nm	267 nm	
				15 fs	20 fs	
				<15 µJ	<5 µJ	
PP2	1030	1000	1800	515 nm	343 nm†	257 nm
				1000 fs		1000 fs
				200 µJ		20 µJ

† Not yet commissioned.

‡ A 50 fs bandwidth-limited option is under preparation.
